# *SNUPN*‐Related Muscular Dystrophy: Novel Phenotypic, Pathological and Functional Protein Insights

**DOI:** 10.1002/acn3.70211

**Published:** 2025-10-06

**Authors:** Nuria Muelas, Pablo Iruzubieta, Alberto Damborenea, Laura Pérez‐Fernández, Inmaculada Azorín, Juan Carlos Jiménez García, Ana Töpf, Pilar Martí, Lorena Fores‐Toribio, María Manterola, Rosana Blanco‐Mañez, Oihane Pikatza‐Menoio, Sonia Alonso‐Martín, Volker Straub, Aitziber L. Cortajarena, Adolfo López de Munain, David De Sancho, Lorea Blázquez, Juan J. Vilchez

**Affiliations:** ^1^ Neuromuscular Diseases Unit, Department of Neurology Hospital Universitari i Politècnic La Fe Valencia Spain; ^2^ Neuromuscular and Ataxias Research Group Instituto de Investigación Sanitaria La Fe Valencia Spain; ^3^ Centro de Investigación Biomédica en Red de Enfermedades Raras (CIBERER) Instituto de Salud Carlos III Madrid Spain; ^4^ Department of Medicine Universitat de València Valencia Spain; ^5^ Department of Neurosciences Biogipuzkoa Health Research Institute San Sebastián Spain; ^6^ Centro de Investigación Biomédica en Red de Enfermedades Neurodegenerativas (CIBERNED) Instituto de Salud Carlos III Madrid Spain; ^7^ Department of Neurology and Neurosurgery Montreal Neurological Hospital and Institute, McGill University Montreal Quebec Canada; ^8^ Center for Cooperative Research in Biomaterials (CIC biomaGUNE) Basque Research and Technology Alliance (BRTA) San Sebastián Spain; ^9^ John Walton Muscular Dystrophy Research Centre Newcastle University and Newcastle Hospitals NHS Foundation Trust Newcastle Upon Tyne UK; ^10^ Electron‐Microscopy Unit, Pathology Department University Hospital La Fe Valencia Spain; ^11^ Stem Cells and Aging Group, Department of Bioengineering, Biogipuzkoa Health Research Institute San Sebastián Spain; ^12^ Ikerbasque Basque Foundation for Science Bilbao Spain; ^13^ Department of Neurology Donostia University Hospital, Osakidetza Basque Health Service San Sebastián Spain; ^14^ Faculty of Medicine University of the Basque Country San Sebastián Spain; ^15^ Faculty of Medicine University of Deusto Bilbao Spain; ^16^ Donostia International Physics Center San Sebastián Spain; ^17^ Faculty of Chemistry University of the Basque Country San Sebastián Spain; ^18^ Institute of Biotechnology and Biomedicine (BIOTECMED) University of Valencia Valencia Spain

**Keywords:** inclusion body myositis (IBM), muscular dystrophy, neurogenetics, SNUPN, snurportin‐1, splicing

## Abstract

**Objective:**

*SNUPN*‐related muscular dystrophy or LGMDR29 is a new entity that covers from a congenital or childhood onset pure muscular dystrophy to more complex phenotypes combining neurodevelopmental features, cataracts, or spinocerebellar ataxia. So far, 12 different variants have been described. Here we report the first family with *SNUPN*‐related muscular dystrophy presenting an adult‐onset myopathy as well as novel ultrastructural findings.

**Methods:**

Clinical evaluation, muscle and brain magnetic resonance imaging (MRI), and muscle histopathological and electron microscopy analysis were conducted. Functional studies including protein modelling and interaction, immunofluorescence and splicing analysis were also performed.

**Results:**

Two siblings carrying two novel deleterious variants in the *SNUPN* gene (p.Arg27Cys and p.Cys174Tyr) showed adult‐onset proximo‐distal and axial muscle weakness with early respiratory involvement. One patient presented with asymptomatic cerebellar atrophy. Muscle MRI identified involvement in the paravertebral, triceps brachii, sartorius and gracilis muscles. The histopathology revealed dystrophic changes and an abnormal pattern of cytoskeletal and myofibrillar proteins, while electron microscopy disclosed the proliferation of granules and vesicles associated with features of nuclear envelope and sarcolemma remodelling. Functional studies showed that *SNUPN* variants impair snurportin‐1 function through reduced binding affinity to importin‐β and impaired folding, leading to disturbed nuclear import of small nuclear ribonucleoproteins and downstream splicing.

**Interpretation:**

Our work expands the phenotype of *SNUPN*‐related muscular dystrophy and provides more insights into their pathological profile. We advise *SNUPN* testing in patients with late‐onset proximo‐distal and axial weakness with early respiratory impairment and features reminding inclusion body myositis (IBM). Granular deposits suggestive of biomolecular condensates perturbed cell organelle traffic and membrane homeostasis, opening new avenues to understand the pathomechanisms involved in this novel disease.

## 1 Introduction

Biallelic variants in the *SNUPN* gene were recently shown to cause a new form of muscular dystrophy varying from a congenital to a childhood‐onset limb girdle muscular dystrophy (LGMDR29, MIM #620793), in some cases accompanied by extramuscular manifestations secondary to central nervous system or ocular involvement [1, 2]. In addition, two families showing spinocerebellar ataxia, mild intellectual disability and muscle weakness have been described [3].

*SNUPN* encodes for snurportin‐1, a protein implicated in the trafficking of small nuclear ribonucleoproteins (snRNPs), essential spliceosome components, from the cytoplasm to the nucleus [4]. Snurportin‐1 deficiency was shown to produce cytoplasmic accumulation of snRNPs, protein aggregates in muscle fibres and a widespread splicing impairment [1, 2]. So far, 12 mutations have been reported; most of them are frameshift or missense variants located in the C‐terminal region of the protein [1, 2, 3].

Here, we report a family carrying two novel missense variants in different regions of *SNUPN* (NM_0011042581; c.79C>T, p.Arg27Cys and c.521G>A, p.Cys174Tyr). These patients have a different and milder phenotype than those previously described, characterised by adult‐onset axial and proximo‐distal limb weakness. Functional studies confirmed snurportin‐1 dysfunction with muscle cytoplasmic accumulation of SmB/B', a snRNP‐specific protein. Also, a splicing impairment pattern similar to the one shown in previously reported LGDMR29 patients was observed. Pathological studies provide further insights into this recently described myopathy. Our study shows that, even if some elements are shared between patients with *SNUPN*‐related myopathy, the phenotype related to this gene is wider than previously reported.

## 2 Material and Methods

### 2.1 Patients and Genetics

Samples and data from the patients included in the study were collected after obtaining informed consent. Research was performed according to international guidelines, and ethical approval was granted by the National Research Ethics Service (NRES) Committee North East–Newcastle & North Tyneside 1 (reference 24/NE/0066). Affected individuals were investigated according to routine clinical standards for neuromuscular diseases, including the muscle biopsy preparation. Whole exome sequencing and analyses were performed through the MYO‐SEQ project [5].

### 2.2 Muscle Biopsy Processing

Samples obtained from deltoid muscle were snap frozen in isopentane chilled in liquid nitrogen and stored at −80°C until needed. Transverse cryo‐sections were processed by routine histological and histochemical techniques, with the following stains: haematoxylin‐eosin (H&E), Gömöri trichrome and adenosine triphosphatase (ATPase) at pH 9.6, 4.6 and 4.3, nicotinamide adenine dinucleotide‐tetrazolium reductase (NADH‐TR), cytochrome oxidase combined with succinate dehydrogenase (COX‐SDH), PAS, alkaline phosphatase and oil‐red‐oil (ORO) were processed following standard procedures [6]. Protein immunolabelling of sarcolemma, cytoskeletal, myofibrillar and inflammatory markers was performed by immunofluorescence as described below.

### 2.3 Immunofluorescence

Frozen muscle sections were fixed in 4% paraformaldehyde (Thermo Scientific) and permeabilised in PBS with 0.5% Triton‐X‐100. Samples were then blocked using a blocking solution [5% bovine serum albumin (BSA), 10% goat serum and 0.025 Tween 20] for 2 h and, afterwards, incubated in primary antibodies overnight at 4°C (Table S1). The day after, three washes in PBS were performed, and fluorescent secondary antibodies (AlexaFluor anti‐mouse 488, anti‐rabbit 555 and anti‐rabbit 647, Invitrogen) were added. Samples were mounted using Fluoromount mounting medium (Thermo Scientific).

### 2.4 Transmission Electron Microscopy

Frozen tissue samples were quickly placed in ice‐cold Trump's fixative and maintained for 2 h, then transferred to a 4°C refrigerator and allowed to fix overnight; following fixation, the tissue was washed with phosphate buffer, post‐fixed in 1% osmium tetroxide, and then processed according to standards [7]. Ultrathin sections were examined with a transmission electron microscope FEI Tecnai Spirit BT (ThermoFisher) provided with a Gatan Orius CCD camera.

### 2.5 Protein Mutational Analysis

The Rosetta modelling suite [8] was employed to estimate free energy changes related to folding and binding. Specifically, the RosettaDDGPrediction [9] Python wrapper was used. For folding free energy differences, the structure predictions for snurportin‐1 (UniprotKB: O95149) from Alphafold 2 [10] and 3 [11] were used as references. For binding predictions, multiple experimental structures, including both the isolated snurportin‐1 importin‐β binding (IBB) domain and its partner, were used. To predict changes in folding free energy due to mutations, the cartddg2020 protocol along with the ref2015 scoring function [12] was applied. This protocol first energetically relaxes the input structure via a Rosetta script. Subsequently, cartesian_ddg is run on the structure with the lowest energy obtained from the relaxation process. The change in free energy is calculated over the free energy differences derived from an ensemble of paired wild‐type and mutated structures. Finally, the predicted impact of mutations is categorised into destabilising, neutral, and stabilising effects. Stabilising mutations are defined by a ΔΔG < −1 kcal/mol, neutral mutations by −1 < ΔΔG < 1 kcal/mol and destabilising mutations by ΔΔG > 1 kcal/mol. For binding predictions, the protocol developed by Barlow and coworkers [13] was used, which estimates changes in binding free energy upon mutation in a protein complex. It applies the ‘backrub’ sampling method [14] to capture local motions observed in crystal structures.

### 2.6 Protein Expression and Purification

Human snurportin‐1 and importin‐β coding sequences were cloned in a pGST‐parallel2 vector modified to include a Tobacco Etch Virus (TEV) protease cleavage sequence between the Glutathione‐S‐transferase (GST) and the protein sequence. All *SNUPN* mutations were introduced using Quick Change mutagenesis and confirmed by Sanger sequencing of the entire open reading frame. Proteins were expressed in *Escherichia coli* strain C41 (DE3) grown in LB medium with 0.1 mg/mL ampicillin. Expression was induced by IPTG when optical density reached 0.5. After 16 h at 18°C, cells were harvested by centrifugation for 15 min at 4500 rpm and resuspended in PBS (pH 7.4) containing 1 mM EDTA and 2 mM DTT. Cells were lysed by sonication, and lysates were centrifuged for 1 h at 10000 rpm. For protein purification, the supernatant was incubated for 16 h at 4°C with Pierce Glutathione Agarose resin (Thermo Scientific). In the case of Importin‐β, the GST‐tag was cleaved by incubation with the Tobacco Etch Virus (TEV) protease overnight at 25°C. All proteins were further purified by gel filtration chromatography using a Superdex 75 10/300 GL column (GE Healthcare) equilibrated in PBS (150 mM NaCl, 50 mM phosphate buffer pH 7.4). Protein concentration was measured by UV absorbance at 280 nm.

### 2.7 In Vitro Pull‐Down Assays

For pull‐down assays, 1 μg of each purified GST‐snurportin‐1 variant was incubated with Pierce Glutathione Magnetic Agarose Beads (Thermo Scientific) for 1 h at room temperature with gentle agitation. Unbound protein was removed, and reactions were incubated with 1 μg Importin‐β for 1 h at room temperature with gentle agitation. Reactions were washed extensively with PBS (pH 7.4) and eluted with 50 mM Tris–HCl containing 10 mM reduced L‐glutathione (pH 8). Eluted fractions were analysed by SDS‐PAGE and visualised by silver staining.

### 2.8 RNA Splicing Analysis

Muscle was homogenised and RNA was isolated as previously described [1]. RNA was reverse transcribed using the High‐Capacity cDNA Reverse Transcription Kit (Applied Biosystems) with random primers following the standard protocol. For RT‐PCR, 10 ng of cDNA was used for each subsequent PCR reaction using 2X Immomix Master Mix and each primer at a final concentration of 0.5 μM. PCR products were visualised in a QIAxcel Advanced System using a QIAxcel DNA Screening kit (QIAGEN). The splicing pattern of alternatively skipped exons was monitored using the oligonucleotides detailed in Table S2.

### 2.9 Statistical Analysis

Statistical analyses were performed using the GraphPad Prism 10. Student's *t*‐test was used when analyzing differences between two groups (Figure 5B). More than two group comparisons were performed with one‐way ANOVA followed by multiple comparisons with a control group (Figure 4D). The alpha level for statistical significance was set at < 0.05.

## 3 Results

### 3.1 Phenotype and Ancillary Tests

**FIGURE 1:**
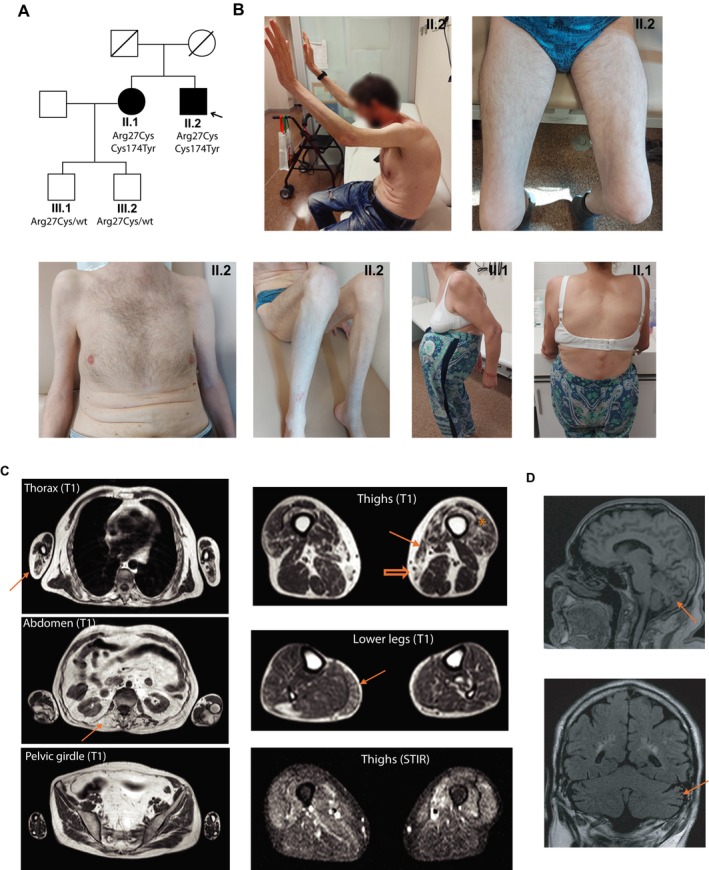
Genetic, clinical, and radiological findings of the family. (A) Pedigree. Patients II.1 and II.2 carried both *SNUPN* variants while the healthy sons of II.1 only carried one of the variants. (B) The proband (II.2) showed axial weakness with camptocormia and diffuse proximal and distal atrophy in upper and lower limbs as well as pectus carinatum. His sister (II.1) also had marked camptocormia and kyphoscoliosis. (C) Muscle MRI from the proband showed involvement of triceps brachii (arrow in thorax), paraspinal muscles (arrow in abdomen), distal portions of vasti (asterisk in thighs), sartorius (arrow in thighs), gracilis (empty arrow in thighs), and muscles of the posterior compartment of the lower legs (arrow in lower legs). Other limb girdle muscles were overall preserved. Focal hyperintensities in vasti and gastrocnemius were detected on T2 weighted STIR sequences. (D) Brain MRI of the proband showed posteroinferior vermian (arrow in the upper image) and bilateral hemisphere cerebellar atrophy (arrow in the lower image).

The proband was a 56‐year‐old male who developed slowly progressive muscular weakness and atrophy involving both proximal and distal muscles of the lower and upper limbs since the age of 41 (Figure 1A,B). He had normal milestones and played sports in his youth. Scoliosis was detected at the age of 12 years, and he wore a brace for 3 years. Since the age of 50, he developed severe camptocormia due to axial weakness and needed a walker to stand up and walk. Mild dysphagia was observed in the last years. At the last examination, no relevant facial weakness was detected. His voice was slightly hypophonic. Diffuse muscle weakness and atrophy of the upper and lower extremities, involving proximal and distal regions including hands (thenar, hypothenar, and first interosseous eminence) was observed. Weakness in triceps brachii muscles was greater than in biceps brachii muscles, and in knee extensors, it was greater than in knee flexors. Also, relevant weakness was observed in neck flexors, trunk, and hip flexors. He had difficulties raising his arms above the horizontal without a clear winged scapula. Kyphoscoliosis and camptocormia were prominent features needing a walker support to stand and walk. Pectus carinatum was also observed (Figure 1B–II.2). Apart from mild finger contractures, no prominent contractures or hyperlaxity were detected. Serum creatine kinase (CK) levels were borderline (285 IU/L). Nerve conduction studies were normal. Overall, a myopathic electromyogram (EMG) pattern was observed, although large motor units were occasionally recorded. No spontaneous activity was detected. Respiratory function tests showed severe restrictive ventilatory dysfunction. On diaphragm ultrasound, excursion amplitude during maximum inspiration, diaphragm thickness at total lung capacity (TLC), and the diaphragm thickening ratio were markedly reduced, revealing diaphragmatic alteration. No cardiac involvement was detected.

Remarkably, his older sister complained of back pain and developed camptocormia in her fifties, needing a walker at 60 years of age. When evaluated at 63 years of age, a similar phenotype to her brother was observed, showing severe trunk and abdominal weakness and orthopnoea (Figure 1B–II.1). CK levels were within normal range. The EMG pattern was myopathic without spontaneous activity. No other family members were affected.

Whole body muscle magnetic resonance imaging (MRI) was performed in the proband. Paravertebral muscles were severely affected as well as the triceps brachii, tensor fasciae latae, sartorius, and gracilis muscles (Figure 1C). The serratus anterior, distal portions of the vasti muscles, and muscles of the posterior compartment of the legs also showed fatty replacement of muscle tissue. Focal signal hyperintensities in the vasti and gastrocnemius muscles were detected on T2‐weighted short tau inversion recovery (STIR) sequences. An axial muscle computed tomography scan performed 9 years later showed progression of fatty transformation, with marked involvement of paraspinal, intercostal, and abdominal muscles (Figure S1A). Moreover, a brain MRI showed posteroinferior vermian and hemisphere cerebellar atrophy (Figure 1D), although no cerebellar signs were found on physical exam.

### 3.2 Histopathological Findings

**FIGURE 2:**
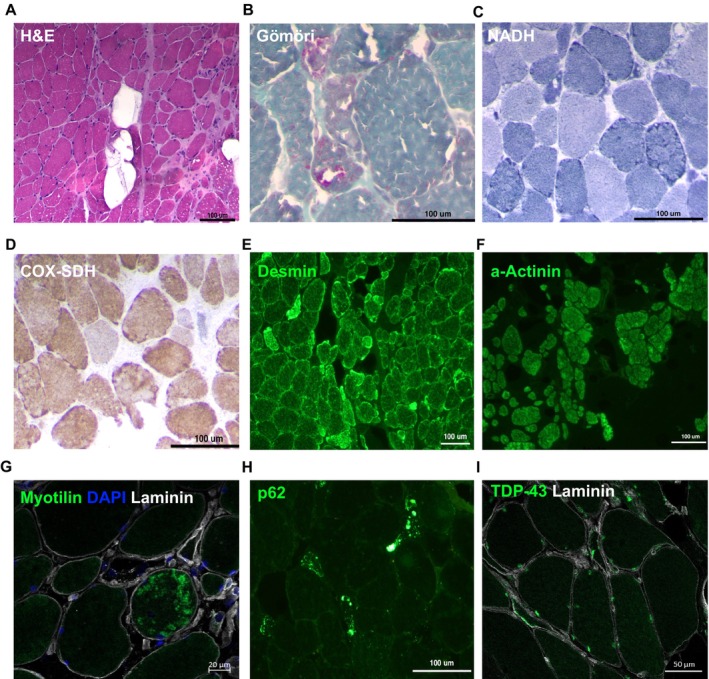
Muscle histopathological findings. (A) Cross‐section stained with Haematoxylin‐eosin (H&E) showing dystrophic features with fibre variability and foci of endomysial fibrosis with necrotic fibres and myophagia. (B) The Gömöri trichrome preparation showed subsarcolemmal and intermyofibrillar granular depositions with occasional rimmed vacuoles. (C, D) Representative images of oxidative enzymes histochemistry (NADH and COX‐SDH) showing irregular reactivity with reinforcements forming trabecular and crescent patterns or hyporeactive zones forming minicore‐like structures. Muscle immunolabelling studies (E–I) show an abnormal pattern with foci of desmin (E), alpha‐Actinin (F), myotilin (G), and p62 (H) protein aggregates in some fibres, while TDP‐43 shows a normal nuclear expression pattern (I).

A deltoid muscle biopsy from the proband showed fibre size variability with hypertrophic and atrophic fibres, and foci of endomysial proliferation of connective tissue with occasional necrotic fibres and inflammatory cell infiltration (Figure 2A, Figure S1B). The atrophic fibres showed rounded contours and rarely appeared with flattened angulated shapes. In addition to nuclei proliferation with partial centralization and occasional empty vacuoles, the most relevant structural features were fine‐coarse intermyofibrillar and subsarcolemmal basophilic (haematoxylin‐eosin) or red (trichrome) granular depositions, occasionally converging amorphous deposits around empty spaces reminiscent of rimmed vacuoles (Figure 2B). Oxidative histochemistry enhanced the subsarcolemmal and intermyofibrillar reinforcements profiling crescents, trabecular or lobulated patterns, and disclosed multi‐minicore non‐reactive areas and frequent COX hyporeactive fibres (Figure 2C,D). The immunolabelling revealed disruptions in the expression pattern of cytoskeleton (desmin) (Figure 2E) and myofibrillar (alpha‐actinin, myotilin) proteins with foci of aggregations (Figure 2F,G). Additionally, scattered fibres showed widespread cytoplasmic punctate or foci of increased expression of the autophagic marker p62 (Figure 2H) while TDP‐43 depicted normal nuclear expression (Figure 2I). Inflammatory markers revealed cell infiltrates of macrophages (CD68) invading necrotic fibres or sparsely spread in endomysium, whereas scattered myofibers showed sarcolemma MHC‐class 1 expression (Figure S1B).

### 3.3 Muscle Transmission Electron Microscopy

**FIGURE 3:**
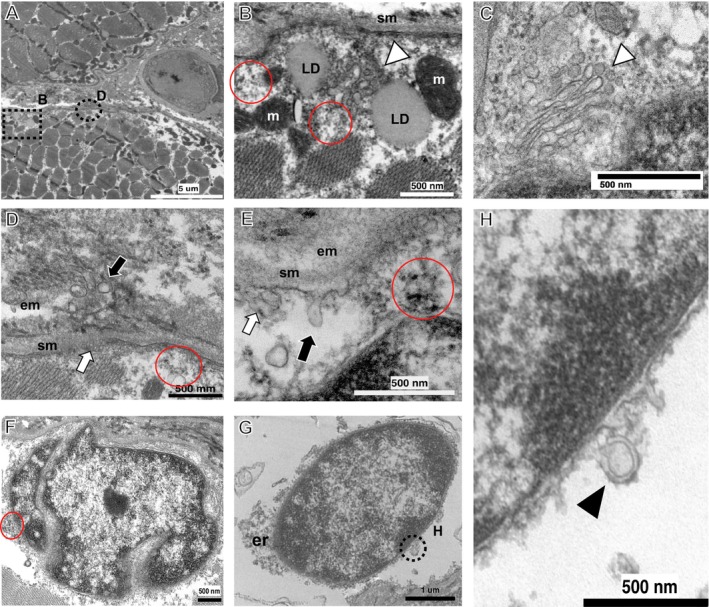
Muscle transmission electron microscopy. Micrograph (A) represents transverse section of two adjacent myofibers separated by a capillary vessel. Note the proliferation of organelles and areas of rarefaction at the subsarcolemmal compartment; the dashed rectangle and circle delimit areas magnified in (B) and (D), respectively. (B) Subsarcolemmal region depicting organelle's collections made up by a core of small, rounded double‐membrane vesicles and tubular or elongated elements compatible with Golgi cisternae (white arrowhead); this core is surrounded by organelles like mitochondria (m) and lipid droplets (LD). Note as well the profuse proliferation of granules possibly featuring biomolecular condensates laying around the different organelles or forming electrondense sarcoplasmic aggregates (red circles). Micrograph (C) shows in detail the sarcoplasmic tubulo‐vesicular arrays forming Golgi cisternae (white arrowhead). Micrographs (D) and (E) depict segments of sarcolemma (sm) presenting invaginations (white arrows); note as well images of exocytic vesicles spelled out into the extracellular matrix (em) in (D) and endocytosis in (E) (white arrows). Micrographs (F–H) represent two subsarcolemmal nuclei with irregular (F) or uniform (G) shapes, surrounded by a cloud of granules and small vesicles more heavily clustered at the poles, probably corresponding to endoplasmic reticulum compartment (er) and electrondense aggregates (red circle). (H) shows a magnified segment of the nuclear envelope membrane (dashed circle in G) depicting abundant anchored granules and numerous membrane outgrowths forming tubular or thorny buds and a double membrane omega pouch (black arrowhead).

Electron microscopy revealed that subsarcolemmal and intermyofibrillar inclusions corresponded to aggregates of mitochondrial arrays with occasional lipid droplets (Figure 3A,B), surrounding clusters of small, rounded double‐membrane vesicles mixed with some ovoid or tubular elements possibly representing Golgi cisternae (Figure 3B,C). The cytosol contained granular particles that could be easily mistaken for glycogen granules but are recognised by their larger size and droplet shape, suggestive of biomolecular condensates (Figure 3B,D–F). At the cell membrane, the inner sarcolemma surface appeared covered with numerous small vesicles, whereas images of exocytic vesicles expelled out into the extracellular matrix (Figure 3D) or endocytosis and multivesicular fusion (Figure 3E) were commonly observed. Nuclei showed variable shapes and chromatin arrangement (Figure 3F,G) and were surrounded by a layer of granular particles and small vesicles heavily clustered around the nuclear poles, possibly corresponding to the endoplasmic reticulum compartment (Figure 3G). Moreover, the nuclear envelope showed profuse outgrowths either as thorny or tubular buds, or double‐membrane omega hernias (Figure 3G,H).

### 3.4 Genetics and Structural Studies

Whole exome sequencing was performed in the proband, which did not reveal any pathogenic or likely pathogenic variants in genes related to myopathies. The exome was re‐assessed after the recent description of *SNUPN*‐related muscular dystrophy, and two variants in *SNUPN* (c.79C>T, p.Arg27Cys, and c.521G>A, p.Cys174Tyr) were identified. Segregation studies showed the variants were in *trans* and segregated with the disease (Figure 1A, Figure S1C). These *SNUPN* variants were present at a very low frequency (0.0003501) for c.79C>T and absent for c.521G>A in gnomAD v.4.1. They affected highly conserved residues across different species (Figure S2A). Moreover, bioinformatic prediction tools, including SIFT (both variants predicted as not tolerated), Polyphen‐2 (1.00 and 0.977), CADD—Combined Annotation Dependent Depletion—score (32 and 26.9), and Alphamissense (0.699 and 0.987) [15] supported the pathogenicity of both variants (Figure S2B–D).

**FIGURE 4:**
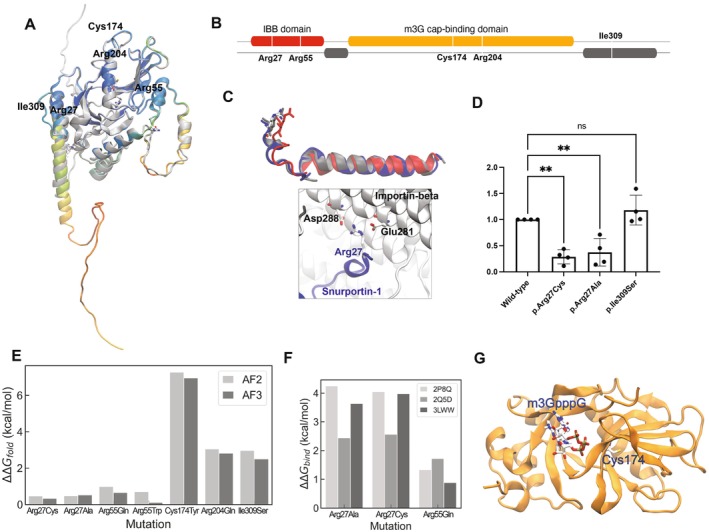
Protein analysis of snurportin‐1 variants. (A) Cartoon representation of the Alphafold 2 (coloured) and 3 (grey) structure of snurportin‐1 highlighting with atomic detail the positions of the mutations. (B) Schematic of the sequence of snurportin‐1, labelled with the amino acids mutated in the reported missense variants. Ordered domains (Interpro: O95149) are highlighted in colour and regions predicted to be disordered (Disprot: DP01874) are shown in grey. (C) The upper panel shows the overlay of three experimental importin‐β‐bound structures of the IBB‐domain (red: 2q5d; blue: 3lww; grey: 2p8q), with Arg27 in atomic detail. The lower panel shows the bi‐dentate salt‐bridge interaction mode of snurportin‐1 (blue) and importin‐β (white). (D) Quantification of 4 independent in vitro pull‐down experiments measuring interaction between recombinant snurportin‐1 and importin‐β (n.s. non‐significant, ** adjusted *p*‐value < 0.01; one‐way ANOVA followed by multiple comparisons with the wild‐type). (E, F) Predicted free energy changes in folding (E) and binding (F) for selected variants using different experimental structures. (G) Cartoon representation of the m3G cap binding domain of snurportin‐1 bound to a m3GpppG‐cap dinucleotide (1 × k5). Both the substrate and Cys174 are shown in atomic detail.

We then investigated the potential effects of p.Arg27Cys and p.Cys174Tyr variants in the structure and function of snurportin‐1. The mutated residues lie in distant regions of the protein and may impair function through different mechanisms (Figure 4A,B). Arg27 lies at the highly conserved region of the importin‐β binding (IBB) domain [16], which also harbors the previously described p.Arg55Gln and p.Arg55Trp variants [2, 3]. In the Alphafold model, the p.Arg27 residue is exposed on the protein surface but forms intramolecular interactions with other residues from the central m3G cap‐binding domain that would be weakened upon mutation. However, the main effect would be in the formation of complexes with importin‐β, where p.Arg27 interacts with negatively charged residues (Glu and Asp) on the inner surface of importin‐β via bidentate salt‐bridge interactions (Figure 4C). This interaction would be depleted in the p.Arg27Cys variant due to the loss of the positive charge, resulting in an obligatory decrease in binding affinity causing a negative impact on the formation of complexes with importin‐β. In line with this model, the p.Arg27Ala mutation has already been shown to disrupt binding to importin‐β, showing a 20‐fold increase in the binding constant (*K*_*d*_) to the IBB domain [17]. Indeed, in vitro pull‐down experiments showed a decreased interaction of recombinant p.Arg27Cys and p.Arg27Ala snurportin‐1 with importin‐β (Figure 4D and Figure S2E). The effects of the mutation were also estimated in both folding and binding. The effects on the folding of p.Arg27 mutations (measured by the folding free energy change: ΔΔ*G*_fold_) were unfavorable but small, as in the case of variants involving Arg55 (Figure 4E). Conversely, the effects on binding (ΔΔ*G*_bind_) were larger than those on folding irrespective of the experimental structure used in our calculations (Figure 4F), indicating a clear decrease in the binding affinity upon the elimination of Arg27. Overall, these results suggest that the p.Arg27Cys variant may result in a disruption of the nuclear import of snRNPs through a reduced interaction of snurportin‐1 with importin‐β.

The second variant, p.Cys174Tyr, is located in the β6 strand, part of the coplanar β‐sheets that shape the binding pocket in the m3G‐cap‐binding domain (Figure 4G). Although the Cys174 residue does not face the binding pocket [18] (Figure 4G), introducing a bulky residue like Tyr may affect protein stability. Indeed, p.Cys174Tyr is the most destabilising variant compared with previously described missense mutations (ΔΔGfold ≈7 kcal/mol, Figure 4E), which is consistent with the high AlphaMissense score (0.987) (Figure S2D).

### 3.5 Functional Studies

**FIGURE 5:**
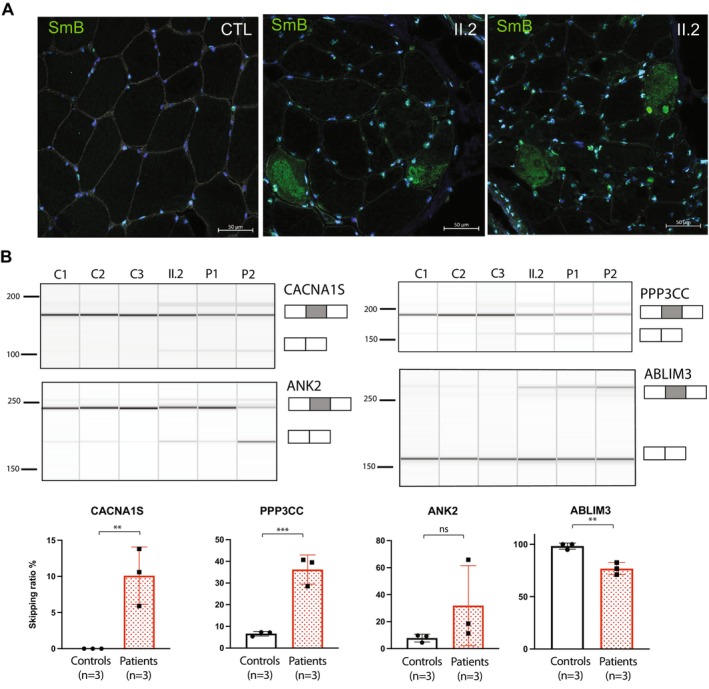
Molecular consequences of *SNUPN* variants in muscle biopsy. (A) Immunofluorescence shows cytoplasmic accumulation of SmB/B′ snRNP specific protein in patient muscle biopsies. (B) RT‐PCR of splicing events in control (C1 and C2) and patients (II.2, P1 and P2) muscle biopsies and their quantification (n.s. non‐significant, **p < 0.01, ***p < 0.001; *t*‐test). II.2 is the proband in this study whereas P1 and P2 correspond to F1.II.2 and F1.II.1 as described in Iruzubieta et al. 2024.

To provide further evidence that *SNUPN* variants were responsible for the myopathy phenotype presented by the patients, we studied molecular alterations previously described in *SNUPN*‐related myopathy. First, we studied SmB/B', a snRNP previously shown to be mislocalised in patients' muscle [1], confirming the presence of cytoplasmic aggregates in the proband (Figure 5A). Furthermore, we analysed the splicing pattern of selected events previously described in the muscle of LGMDR29 patients by RT‐PCR (Figure 5B) [1]. Abnormal splicing was confirmed in four events present in genes encoding relevant proteins for muscle function (*CACNA1S*, *PPP3CC*, *ANK2*, and *ABLIM3*). Aberrant splicing pattern was detected in the proband and confirmed in two patients from a previously described family with LGMDR29 [1]. These findings further support splicing impairment in patients with *SNUPN*‐myopathy.

## 4 Discussion

Here, we describe two patients from the same family showing an adult‐onset axial and limb proximo‐distal myopathy due to novel biallelic variants in the *SNUPN* gene, expanding the clinical spectrum of *SNUPN‐*related myopathy or LGMDR29. In contrast to previously described phenotypes [1, 2, 3], these individuals showed a myopathy with later onset and milder severity, borderline CK levels, and absent joint contractures. However, they shared common features with previously reported patients, including early respiratory failure and a similar pattern of muscle involvement in MRI, highlighting the impairment of the sartorius and gracilis muscles. Furthermore, the proband's muscle biopsy recapitulated many findings previously reported in LGMDR29 patients, including dystrophic changes, disarrangement of the intermyofibrillar and subsarcolemmal structures, and irregular expression with focal aggregates of scaffold proteins of the cytoskeleton and Z‐line. Interestingly, in line with muscle MRI STIR hyperintensities, the biopsy showed inflammatory changes that, although represent a common feature within the dystrophic profile, have not been explicitly documented previously.

The ultrastructural analysis in the proband revealed novel insights. The deposits of sarcoplasmic granules may represent condensed molecular complexes of nucleic acids and ribonucleoproteins that change from liquid–liquid soluble phases (LLP) to an insoluble state [19]. Stress granules induced by cellular stress are the best‐known examples [20], but there are many complexes with LLP attributes playing diverse roles in cellular processes such as the interaction between nucleoporins and nucleocytoplasmic transport receptors [21], adaptors and membrane receptors regulating vesicular transport and sorting [22], and membrane‐less organelles specialised in RNA metabolism or in the biogenesis of neural synapses [19]. Future research will be needed to elucidate the role of mutated snurportin‐1 in these sarcoplasmic granular deposits.

Another remarkable finding concerns to the active remodelling of the nuclear envelope and sarcolemma membranes involving exocytosis, endocytosis, and vesicular fusion. Such profiles, along with extensive subsarcolemmal vesicular proliferation, may indicate upregulation of membrane repair processes [23]. Remarkably, many muscular dystrophies are caused by proteins involved in sarcolemma defects or membrane repair machinery [24, 25]. Indeed, an abnormal expression of proteins of the dystrophin‐associated glycoprotein complex, relevant for sarcolemma maintenance, has been reported in muscle biopsies from *SNUPN* patients [2]. Furthermore, although nuclear envelope abnormalities are commonly observed in diverse myopathies [26], specific features of autophagic remodelling like those observed in this study (e.g., omega buddings) have been scarcely documented in human disorders. However, they have been described in detail in experimental models with variants in nucleoporin genes causative of neurodevelopmental disorders [27].

Our observations suggest that mutated snurportin‐1 and the consequent cytoplasmic accumulation of snRNPs may form biomolecular condensates that perturb nuclear pore transit, organelles, and signalling traffic and interfere with sarcolemma and nuclear envelope maintenance [21, 22]. Cellular autophagic mechanisms would promote the removal of insoluble granules and membrane damage via endocytic pathways leading to lysosomal degradation or recycling [28]. The increased p62 expression in the proband's muscle biopsy indicates endo‐lysosomal upregulation, but further studies that explore these pathways [20] are required. Moreover, our ultrastructural findings need to be documented in additional patients with *SNUPN*‐related disease, as these could represent specific features of this individual.

Protein structural studies supported the pathogenicity of the variants reported here. Hence, p.Arg27Cys altered snurportin‐importin β binding, and p.Cys174Tyr was predicted to have a severe destabilising effect. At a functional level, snurportin‐1 impairment was shown by the aggregation of SmB/B′ protein and an aberrant splicing pattern in the proband's muscle, which was originally described in patients with *SNUPN*‐related muscular dystrophy [1].

The phenotype of these patients raises a differential diagnosis with other myopathies presenting early respiratory failure like HMERF (caused by *TTN* variants) [29], *COL6*‐related myopathies [30], thymidine kinase 2 deficiency (TK2d) [31, 32], or late‐onset Pompe disease [33]. Inclusion body myositis (IBM) should also be considered in the differential diagnosis, given the clinical phenotype with distal hand and knee extensor weakness, quadriceps fatty replacement in MRI, low CK level, as well as inflammatory infiltrates, rimmed vacuoles, increased MHC‐1 expression, and abundant COX‐negative myofibers [34]. However, in patients with *SNUPN*‐muscular dystrophy, TDP‐43 was normally localised in myonuclei (Figure 2I).

Another disease to consider is LGMDD2, caused by heterozygous frameshift mutations in *TNPO3*, which encodes transportin‐3 [35], a protein of the importin‐β family [36]. This is especially interesting because both snurportin‐1 and transportin‐3 are involved in nuclear‐cytoplasmic transport. Additionally, LGMDR29 and LGMDD2 share characteristic markers, such as a predominant involvement of sartorius and gracilis muscles in MRI, and a muscle biopsy with dystrophic features, cytoskeletal and myofibrillar disarrangement, protein aggregates, and upregulated autophagia [35, 37, 38]. Furthermore, both disorders present abnormalities in splicing [39]. LGMDD2 also manifests a broad clinical spectrum, including congenital hypotonia, childhood onset with severe contractures and scoliosis, milder late‐onset phenotypes, and asymptomatic non‐penetrant forms [35], although it follows a dominant transmission pattern [35, 40]. Remarkably, the description of a milder and late‐onset phenotype in a *SNUPN*‐related family increases the similarities between these two disorders.

Current evidence suggests that the location (N‐ or C‐terminal) and the type of variant (missense vs. frameshift) might be relevant for the clinical variability observed in patients with *SNUPN‐*related disorders. Most of the reported variants showing a congenital or childhood onset disease are located in the C‐terminal region of the protein [1, 2, 3]. Therefore, the milder phenotype in this family could be explained because their variants are missense and do not lie in the C‐terminal region of snurportin‐1. However, even patients from the same family carrying the same variants show significant variability in muscular weakness and disease severity, suggesting additional genetic and/or environmental modifying factors might be involved [1].

In conclusion, *SNUPN* variants can produce a broader disease spectrum than previously reported. Here we present the mildest phenotype described so far. Future studies will likely describe additional phenotypes and provide a better understanding of the full spectrum of this disease and the factors that influence its clinical variability. Finally, we uncover new pathological insights, including features of sarcoplasmic granules, upregulated autophagy, nuclear and sarcoplasmic membrane remodelling, and impaired vesicular traffic.

## Author Contributions

N.M., P.I., S.A.‐M., V.S., A.L.C., A.L.M., D.D.S., L.B. and J.J.V. contributed to the conceptualization of this study. P.I., A.D., L.P.‐F., I.A., J.C.J.G., A.T., P.M., L.F.‐T., M.M., R.B.‐M. and O.P.‐M. performed the experiments and statistical analysis. N.M., P.I., L.B. and J.J.V. drafted the initial manuscript. All authors then provided important intellectual input into the final manuscript.

## Consent

Research was performed according to international guidelines, and ethical approval was granted by the National Research Ethics Service (NRES) Committee North East–Newcastle and North Tyneside 1 (reference 24/NE/0066).

## Conflicts of Interest

The authors declare no conflicts of interest.

## Supporting information


**Data S1:** acn370211‐sup‐0001‐Tables.pdf.

**Figure S1:** acn370211‐sup‐0002‐FigureS1.pdf.

**Figure S2:** acn370211‐sup‐0003‐FigureS2.pdf.

## Data Availability

The data that support the findings of this study are available on request from the corresponding author. The data are not publicly available due to privacy or ethical restrictions.
