# Supplementary material for: SNUPN‐Related Muscular Dystrophy: Novel Phenotypic, Pathological and Functional Protein Insights
Source: Ann Clin Transl Neurol. 2025 Oct 6;13(2):285–95. doi: 10.1002/acn3.70211 (PMC12883674; doi:10.1002/acn3.70211)
Supplement: Supplementary file 1 — Data S1: acn370211‐sup‐0001‐Tables.pdf. [file ACN3-13-285-s002.pdf]

## Supplementary Table 1: antibodies

| Name | Company | Reference | Host | Dilution |
|---|---|---|---|---|
| Alpha actinin (sarcomeric) | Sigma-Aldrich | A7732 | Mouse | 1/100 |
| Desmin | Abcam | ab15200 | Rabbit | 1/200 |
| SQSTM1/P62 | Abcam | ab56416 | Mouse | 1/100 |
| CD68 | Santa Cruz Biotechnology | sc-20060 | Mouse | 1/200 |
| MHC class I | Santa Cruz Biotechnology | sc-32235 | Mouse | 1/50 |
| Myotilin | Leica Biosystems | NCL-MYOTILIN | Mouse | 1/100 |
| SmB/B’ | Santa Cruz Biotechnology | sc-374009 | Mouse | 1/100 |
| Laminin | Sigma-Aldrich | L9393 | Rabbit | 1/100 |

## Supplementary Table 2: RT-PCR oligonucleotides

| Name | Company | Sequence | Description |
|---|---|---|---|
| ABLIM3 Fw | Merck-Sigma | CTACGAGAACCTGGACCTCC | Exon 14 skipping 178 nt, inclusion 277 nt (NM_014945.5). |
| ABLIM3 Rv | Merck-Sigma | GGGGTTTCCGGTAGATGTTAC | |
| ANK2 Fw | Merck-Sigma | TGAGACGATGACTGAGGTTCT | Exon 24 skipping 192 nt, inclusion 246 nt (NM_001386173.1). |
| ANK2 Rv | Merck-Sigma | CTGTCAGATCGTCCTCCCTC | |
| CACNA1S Fw | Merck-Sigma | CCTGATTGTCATTGGCAGCA | Exon 29 skipping 111 nt, inclusion 168 nt (NM_000069). |
| CACNA1S Rv | Merck-Sigma | AGCCTCATGACACGGAACAG | |
| PPP3CC Fw | Merck-Sigma | GCAAGAAAGTGAGAGTGTGCT | Exon 13 skipping 160 nt, inclusion 190 nt (NM_005605.5). |
| PPP3CC Rv | Merck-Sigma | CCTCGCGCTTCTTCAAAACT | |
