# Supplementary material for: SNUPN‐Related Muscular Dystrophy: Novel Phenotypic, Pathological and Functional Protein Insights
Source: Ann Clin Transl Neurol. 2025 Oct 6;13(2):285–95. doi: 10.1002/acn3.70211 (PMC12883674; doi:10.1002/acn3.70211)
Supplement: Supplementary file 2 — Figure S1: acn370211‐sup‐0002‐FigureS1.pdf. [file ACN3-13-285-s001.pdf]

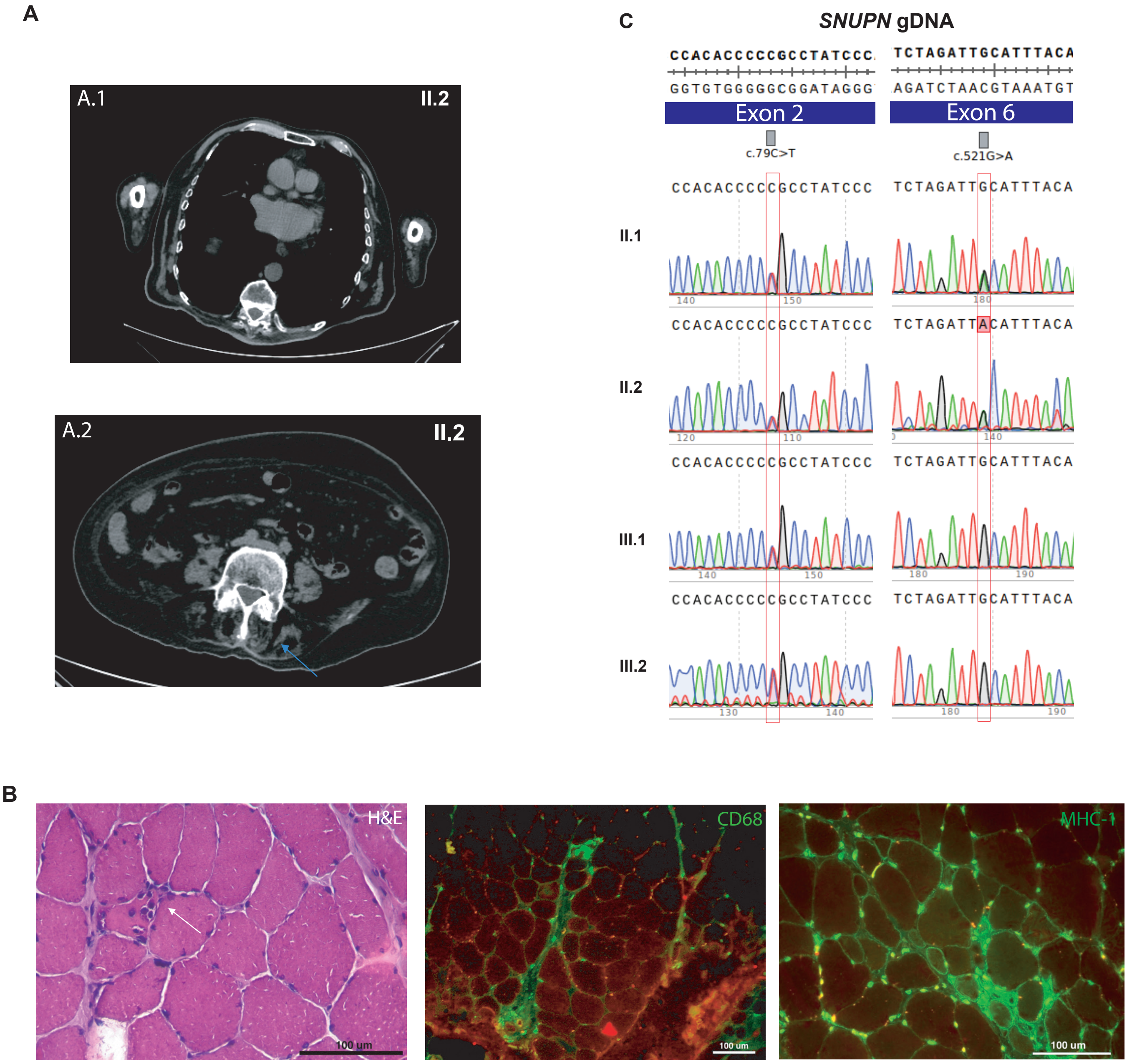

Supplementary Figure 1: Related to Figures 1 and 2: A) Computed tomography of the proband (patient II.2) showing severe involvement of paraspinal (arrow), intercostal and abdominal muscles. B) Some inflammation was identified in myofagic foci positive for the macrophage marker CD68 and scattered fibers expressing MHC-1. C) Sanger sequencing electropherograms in genomic DNA show segregation of *SNUPN* variants in the family under study.
