# Supplementary material for: SNUPN‐Related Muscular Dystrophy: Novel Phenotypic, Pathological and Functional Protein Insights
Source: Ann Clin Transl Neurol. 2025 Oct 6;13(2):285–95. doi: 10.1002/acn3.70211 (PMC12883674; doi:10.1002/acn3.70211)
Supplement: Supplementary file 3 — Figure S2: acn370211‐sup‐0003‐FigureS2.pdf. [file ACN3-13-285-s003.pdf]

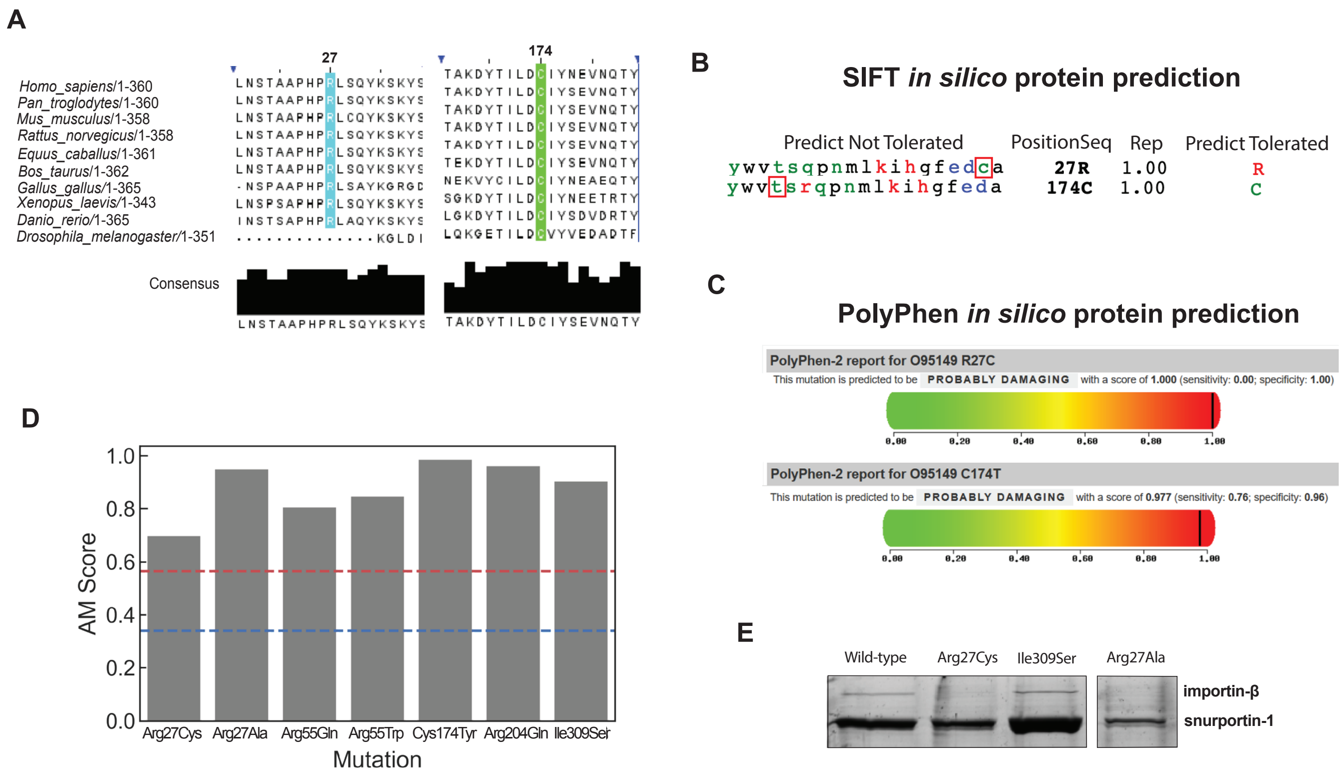


**Supplementary Figure 2:** A. Protein alignment and aminoacid conservation of mutated residues. B-C. *In silico* protein predictions of snurportin-1 variants in Polyphen (B) and SIFT (C) protein prediction servers. D. Alpha Missense scores for missense variants in snurportin-1. The blue and red dashed lines of AM scores mark the thresholds for likely benign and likely pathogenic mutations, respectively. E. Representative gel image from an *in vitro* pull-down experiment between recombinant snurportin-1 variants (lower band) and importin-β (upper band). All lanes are coming from the same replicate experiment although they are shown in separate sections as they were not loaded in the gel in consecutive order.
